# Whole Genome Sequence of Bacillus anthracis MDMC_159, Isolated from the Moroccan Desert Erg Lihoudi 

**DOI:** 10.1128/mra.01209-22

**Published:** 2023-02-13

**Authors:** Amina Manni, Mariem laamarti, Salah Eddine Azaroual, Issam Meftah Kadmiri, Rachid Daoud, Achraf El Allali, Abdelkarim Filali-Maltouf

**Affiliations:** a Department of Biology, Laboratory of Microbiology and Molecular Biology, Mohammed V University, Rabat, Morocco; b Plant and Microbial Biotechnology Center, Moroccan Foundation for Advanced Science, Innovation, and Research, Rabat Design Center, Rabat, Morocco; c African Genome Center, Mohammed VI Polytechnic University, Ben Guerir, Morocco; d Microbiome, AgroBioSciences Mohammed VI Polytechnic University, Ben Guerir, Morocco; University of Rochester School of Medicine and Dentistry

## Abstract

We report the complete genome sequence of Bacillus anthracis strain MDMC_159, which was isolated from a deserted sandy area of Erg Lihoudi in southeastern Morocco. This strain is pathogenic and well adapted to the harsh conditions of the Moroccan desert.

## ANNOUNCEMENT

Bacillus anthracis strain MDMC_159 was isolated from nonvegetated sand dunes from the Erg Lihoudi region in southeastern Morocco (29°54′07.6″N, 5°41′12.1″W) (1). Soil samples were collected from a depth of 0 to 5 cm and suspended in 9 mL physiologic water for a 1-g soil sample. Serial dilutions were made by adding 1 mL of soil suspension to the next tube in the series, up to 10^−7^ dilution; 0.1-mL aliquots from 10^−3^ to 10^−7^ dilutions were spread on nutrient broth (NB) agar plates (Merck). Bacterial samples were cultured, incubated overnight at 28°C, and then stored in 15% glycerol at −80°C.

Genomic DNA extraction was performed from distinct purified colonies of bacteria using the PureLink genomic DNA minikit (K182001; Invitrogen) according to the manufacturer’s instructions. Extraction yield was measured with a fluorometric assay using the high-sensitivity (HS) double-stranded DNA (dsDNA) assay kit in a Qubit 4.0 fluorometer (Invitrogen). The 16S rRNA gene was amplified using the universal primers FD1 and RS16. We sequenced the PCR products at the Pasteur Institute (Lille, France) using a 3730xl DNA Sanger sequencer. The BLASTn analysis of the 16S RNA gene sequence obtained confirmed that the isolate belonged to the *Bacillus* genus. Whole-genome sequencing was performed on the Oxford Nanopore Technologies MinION platform, using a rapid barcoding kit (SQK-RBK004). The long-read library was constructed with 400 ng of total genomic DNA, and a cleanup step was performed using Agencourt AMPure XP beads to remove short fragments. Sequencing of the prepared library was carried out using a MinION Mk1b sequencer (MinION flow cell R9.4.1), and raw sequencing data were base called and analyzed using the Guppy base caller v5.1.13 (2) and MinKNOW v4.5.4. It is important to note that, despite being a strong tool for DNA sequencing, Nanopore sequencing has certain limitations, compared to other technologies, including its poorer accuracy and low throughput (3).

The quality of the sequencing data was assessed using FastQC (4). Trimming and correction of the sequence were performed using Trimmomatic v0.39 (5). The assembly was performed using Flye v2.9 (https://github.com/fenderglass/Flye), and the polishing and preprocessing were performed using Racon (6). The quality of the genome assembly was assessed using QUAST v5.0.2 (7). Annotation was performed with the NCBI Prokaryotic Genome Annotation Pipeline (PGAP) v5.3 (https://www.ncbi.nlm.nih.gov/genome/annotation_prok). The sequence homology among genomes in the same Bacillus cereus group was evaluated using the Genome-to-Genome Distance Calculator (GGDC) v3.0, a digital DNA-DNA hybridization (dDDH) tool (8). Default parameters were used for all of the aforementioned software tools.

The TYGS assigned strain MDMC_159 to the species Bacillus anthracis, with a dDDH value of 81.80% (79% to 84.4%). Additional genetic data using NCBI annotation results are summarized in Table 1.

**TABLE 1:** Genomic features of strain MDMC_159

Feature^*a*^	Finding for B. anthracis strain MDMC_159
No. of reads	43,421
No. of contigs	6
Genome coverage (×)	86
Contig *N*_50_ (bp)	5,185,090
Total sequence length (bp)	5,709,902
GC content (%)	35.5
No. of CDSs	3,025
No. of rRNAs	42
No. of tRNAs	105
No. of tmRNAs	1
No. of ncRNAs	5
No. of pseudogenes	2,906

a CDSs, coding DNA sequences; tmRNAs, transfer-messenger RNAs; ncRNAs, noncoding RNAs.

### Data availability.

The complete genome sequence and annotation data for strain B. anthracis strain MDMC_159 were deposited in GenBank under accession number JAPDEE010000000. The associated BioProject and BioSample accession numbers are PRJNA894224 and SAMN31445157, respectively. The Sequence Read Archive (SRA) accession number is SRX18051163. The 16S rRNA gene sequence was submitted to GenBank under accession number OP703662.
